# Chapin A. Harris, A. M., M. D., D. D. S.

**Published:** 1895-06

**Authors:** 


### Editorial, Etc.

CHAPIN A. HARRIS A. M., M. D., D. D. S.—During the past twenty years several attempts have been made to perpetuate the memory of this truly great and useful man, by some visible mark of appreciation on the part of the dental profession of the United States for the valuable services he rendered to the art and science of dentistry during his professional career in the city of Baltimore.

Such attempts, owing to various causes, came to naught and a movement has again arisen which, there is every reason for believing, promises to be more successful than any previous one, and which the following circular explains:

### CHAPIN A. HARRIS, MEMORIAL FUND.

BALTIMORE, May 1895.

*To the Dentists of the United States:*—

A movement is now in progress to insure the mortuary tribute to the memory of one of the greatest names in the annals of Dentistry. It is proposed to adorn his neglected tomb at Mount Olivet Cemetery with a monumental testimonial worthy of his name and fame.

Mr. Ernest W. Keyser, the talented Baltimore sculptor now in Paris, has modelled a remarkably faithful portrait bust of Dr. Harris and executed designs intended for a monument to be erected over his grave.

It is also in contemplation that should sufficient funds be realized a memorial tablet containing an alto relievo bronze bust of Dr. Harris be placed in both the Baltimore College of Dental Surgery and the Dental Department of the University of Maryland.

It is believed that the necessary funds can be obtained through voluntary subscriptions from the Dental Profession. Snowden & Cowman Manufacturing Company of Baltimore has kindly consented to act as custodians of the "Harris Memorial fund." Contributions are earnestly solicited and will be thankfully acknowledged.

Address: SNOWDEN & COWMAN M'F'G. Co,
9 West Fayette Street,
Baltimore, Md.

Dr. Chapin A. Harris was born in 1806 at Pompey, Onandaga County, New, York. He studied medicine, removed to Ohio and afterwards settled in Baltimore, Md., where he practiced Dentistry until his death in 1860. He founded and assisted in the organization of the first Dental College in the world—"The Baltimore College of Dental Surgery"—chartered in 1839. Was for many years its Professor of Dental Surgery. He edited the AMERICAN JOURNAL OF DENTAL SCIENCE, the first of its kind ever published, from 1839 until 1860, the year in which he died—and was a voluminous contributor to other Dental and Medical Journals. He was the author of "Principles and Practice of Dental Surgery," "Characteristics of the Human Teeth," Diseases of the Maxillary Sinus," "Harris' Dental Dictionary," and edited Fox's Natural History and Diseases of the Human Teeth," with additions, etc. These books were and are widely known to the civilized world.

In 1892 Dr. S. J. Cockerille, of Washington, D. C., delivered an address before the Virginia State Dental Society, from which the following excerpts are quoted: "The time arrived when dentistry demanded an honorable place among her sister sciences, and the Baltimore Dental College sprang into existence as the natural fruit of Harris' earnest and protracted labors." "He was the first to teach that the attainment of excellence in all things connected with dentistry is not only a high moral and personal duty but is likewise an obligation imposed by the community whose bounty is enjoyed." "Considered simply as a dentist Harris was a genius." "He devoted his life to the earnest study of medical and scientific literature." "It would be difficult to overestimate the value of the services which he rendered to the cause of higher education. Every lover of dentistry owes a distinct debt of gratitude to the man." "His rule of conduct was to keep his mind unbiased by theories and speculations; never to have any wish that an investigation should result in favor of this view in preference to that, and never to attempt by premature speculation to anticipate the result of investigations but always to trust to the investigations themselves." "Harris was a hard worker and devoted all the resources of his cultivated mind to further the welfare of his profession." "With the students he was punctual, truthful, straightforward, industrious and always kind." "In his family he was forbearing and loving to his children; to his wife he was the perfection of purity and devotion." "He was more than a dentist—he was a Christian gentleman."

"Honor his memory; keep it green in your hearts and hand it down to your children and children's children to the remotest generations, making the profession a blessing to them." "We expected the great State of New York to step forward and do justice to her noblest son; she failing, we turned with anxious eye to his adopted State, the state that gave to the world its first Dental College, but we have waited in vain." "Let not the names of our great and useful men pass into oblivion."

From the manner in which this commendable effort of 1895 has already been received we feel confident that it will be successful, and we earnestly call upon the members of a profession to which Chapin A. Harris was so warmly devoted, and for which he did so much during the greater part of his earthly career, to aid in this movement by contributing according to their ability. It would require but a small sum from each of the thousands of practitioners of dentistry in this country, all of whom must acknowledge that to Chapin A. Harris is due the honor of having been among the first to rescue dentistry from the condition of a mere trade, and to elevate it to its present status. Let all of us, therefore, unite in preserving the mortal remains of Chapin A. Harris from oblivion, and honor his memory by placing enduring tablets in the two Dental Colleges of Baltimore, one of which was established by his individual efforts, and the other through his teachings.

